# Hexose-6-phosphate Dehydrogenase Modulates 11β-Hydroxysteroid Dehydrogenase Type 1-Dependent Metabolism of 7-keto- and 7β-hydroxy-neurosteroids

**DOI:** 10.1371/journal.pone.0000561

**Published:** 2007-06-27

**Authors:** Lyubomir G. Nashev, Charlie Chandsawangbhuwana, Zoltan Balazs, Atanas G. Atanasov, Bernhard Dick, Felix J. Frey, Michael E. Baker, Alex Odermatt

**Affiliations:** 1 Institute of Molecular and Systems Toxicology, University of Basel, Basel, Switzerland; 2 Department of Nephrology and Hypertension, University of Berne, Berne, Switzerland; 3 Department of Medicine, University of California, San Diego, La Jolla, California, United States of America; 4 Division of Immunopathology, Institute of Pathology, University of Berne, Berne, Switzerland; Cairo University, Egypt

## Abstract

**Background:**

The role of 11β-hydroxysteroid dehydrogenase type 1 (11β-HSD1) in the regulation of energy metabolism and immune system by locally reactivating glucocorticoids has been extensively studied. Experiments determining initial rates of enzyme activity revealed that 11β-HSD1 can catalyze both the reductase and the dehydrogenase reaction in cell lysates, whereas it predominantly catalyzes the reduction of cortisone to cortisol in intact cells that also express hexose-6-phosphate dehydrogenase (H6PDH), which provides cofactor NADPH. Besides its role in glucocorticoid metabolism, there is evidence that 11β-HSD1 is involved in the metabolism of 7-keto- and 7-hydroxy-steroids; however the impact of H6PDH on this alternative function of 11β-HSD1 has not been assessed.

**Methodology:**

We investigated the 11β-HSD1-dependent metabolism of the neurosteroids 7-keto-, 7α-hydroxy- and 7β-hydroxy-dehydroepiandrosterone (DHEA) and 7-keto- and 7β-hydroxy-pregnenolone, respectively, in the absence or presence of H6PDH in intact cells. 3D-structural modeling was applied to study the binding of ligands in 11β-HSD1.

**Principal Findings:**

We demonstrated that 11β-HSD1 functions in a reversible way and efficiently catalyzed the interconversion of these 7-keto- and 7-hydroxy-neurosteroids in intact cells. In the presence of H6PDH, 11β-HSD1 predominantly converted 7-keto-DHEA and 7-ketopregnenolone into their corresponding 7β-hydroxy metabolites, indicating a role for H6PDH and 11β-HSD1 in the local generation of 7β-hydroxy-neurosteroids. 3D-structural modeling offered an explanation for the preferred formation of 7β-hydroxy-neurosteroids.

**Conclusions:**

Our results from experiments determining the steady state concentrations of glucocorticoids or 7-oxygenated neurosteroids suggested that the equilibrium between cortisone and cortisol and between 7-keto- and 7-hydroxy-neurosteroids is regulated by 11β-HSD1 and greatly depends on the coexpression with H6PDH. Thus, the impact of H6PDH on 11β-HSD1 activity has to be considered for understanding both glucocorticoid and neurosteroid action in different tissues.

## Introduction

Originally, 11β-HSD1 was identified in a search for a dehydrogenase catalyzing the conversion of active 11β-hydroxyglucocorticoids (cortisol and corticosterone) into inactive 11-ketoglucocorticoids (cortisone and 11-dehydrocorticosterone)[1]. In lyzed cells and upon purification, 11β-HSD1 catalyzes both dehydrogenase and reductase reaction [2], [3], whereas it acts predominantly as a reductase in intact hepatocytes and differentiated adipocytes [4]–[7]. Recent studies suggested that *in vivo* hexose-6-phosphate dehydrogenase (H6PDH) generates NADPH in the endoplasmic reticulum (ER) lumen, which causes 11β-HSD1 to function as a reductase [5]. Evidence for this role for H6PDH in the catalytic specificity of 11β-HSD1 comes from several studies. In preadipocytes with low expression of H6PDH, 11β-HSD1 was found to be primarily a dehydrogenase, whereas in differentiated adipocytes with high H6PDH expression, 11β-HSD1 was a reductase [5], [8]. Importantly, mice lacking H6PDH are unable to reduce 11-dehydrocorticosterone to corticosterone [9].

H6PDH colocalizes with 11β-HSD1 at the lumenal surface of the ER-membrane [10], where H6PDH provides cosubstrate NADPH and strongly stimulates 11β-HSD1 reductase activity [10]–[14]. Thus, a tightly controlled ratio of H6PDH to 11β-HSD1 is crucial since the local regeneration of cortisol and corticosterone in tissues such as liver, skeletal muscle and adipose tissue determines the magnitude of glucocorticoid receptor (GR) activation and subsequent regulation of genes involved in carbohydrate and lipid metabolism. Indeed, chronically elevated local reactivation of glucocorticoids by enhanced 11β-HSD1 activity has been associated with the development of the metabolic syndrome [15]. Although it has not been analyzed so far, modulation by H6PDH might also be relevant for alternative functions of 11β-HSD1, including the detoxification of xenobiotics [16] and the metabolism of 7-keto- and 7-hydroxy-sterols and -steroids [6], [17]–[19].

Recently, it has become clear that 11β-HSD1 also can catalyze the interconversion of the C7-ketone and the C7-hydroxyl on DHEA [19]–[22]. DHEA is synthesized in the adrenal glands and serves as a major sex steroid hormone precursor [23]. Besides, another important site for synthesis of DHEA is the brain, where it is further metabolized to its 7α- and 7β-hydroxy-forms. DHEA and its C7-oxygenated metabolites are considered neurosteroids and they play a role in the regulation of emotional responses, memory functions and neuronal excitability [24]–[26]. Pregnenolone and its metabolites also are neurosteroids. 7α-hydroxypregnenolone is the major neurosteroid in amphibians, increasing the neuron locomotor activity in the breeding newts [27].

The major metabolic fate of DHEA and pregnenolone in the brain and other extrahepatic tissues is the CYP7B1-dependent 7α-hydroxylation. It was suggested that 7α-hydroxy-DHEA serves as a precursor for other active derivatives [28]. A causal link between declining DHEA levels and age-related loss of cognitive function, including Alzheimer's disease, has been proposed, with evidence for reduced hippocampal CYP7B1 expression [29], [30]. Moreover, several studies suggest that 7-keto- and 7-hydroxy-steroids exert neuroprotective, anti-glucocorticoid and immune-modulatory effects [19], [29], [31], [32]. The presence of 11β-HSD1 activity in the brain suggests that 11β-HSD1 may have a role in regulating the neurosteroid actions of the 7-keto- and 7-hydroxy-metabolites of DHEA and pregnenolone. However, details of the role of H6PDH in their metabolism and their *in vivo* regulation remained unclear, and the reaction direction of the metabolism of these compounds has not been assessed.

Here, we employed enzyme activity measurements and 3D-structural modelling to investigate how 7-keto-, 7α-hydroxy- and 7β-hydroxy-DHEA interact with 11β-HSD1. Furthermore, we determined the impact of H6PDH on the 11β-HSD1-dependent metabolism of various 7-keto- and 7-hydroxy-steroids in intact cells by measuring both initial rates of activity and steady state concentrations. We find that when H6PDH supplies sufficient NADPH, this results in 11β-HSD1 having a preference for the reduction of 7-keto-DHEA to 7β-hydroxy-DHEA. Thus, *in vivo*, depending on the level of H6PDH activity, 11β-HSD1 can regulate the equilibrium between 7-keto-, 7α-hydroxy- and 7β-hydroxy-DHEA in brain and other organs.

## Results

### 11β-HSD1 catalyzes the interconversion of 7-keto- and 7-hydroxy-DHEA

To determine the ability of 11β-HSD1 to catalyze the interconversion of 7-keto- and 7-hydroxy-DHEA in intact cells, we employed HEK-293 cells that were transfected with either 11β-HSD1 alone or together with H6PDH. This cell line is suitable to assess the effect of H6PDH on 11β-HSD1 function, because of low endogenous H6PDH and lacking 11β-HSD1 and 11β-HSD2 expression, which is indicated by Ct values that were higher than 32 [10].

**Figure 1:**
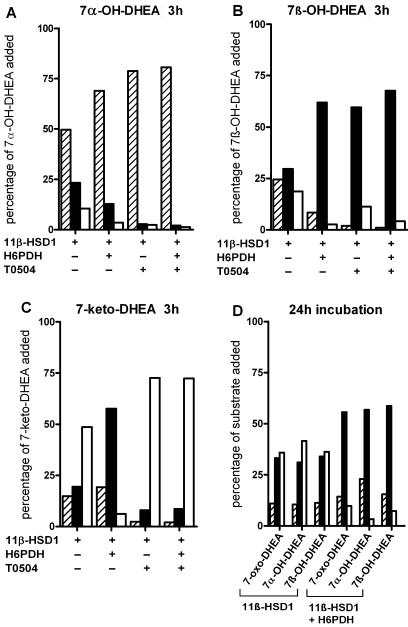
Interconversion of 7α-hydroxy-, 7β-hydroxy- and 7-keto-DHEA by 11β-HSD1 in intact cells. HEK-293 cells transfected with a plasmid for 11β-HSD1 and either a control vector or a plasmid for H6PDH were incubated with 1 µM of 7α-hydroxy-DHEA (7α-OH-DHEA) (*A*), 7β-hydroxy-DHEA (7β-OH-DHEA) (*B*) or 7-keto-DHEA (*C*) in absence or presence of 11β-HSD1 inhibitor T0504, followed by determination of C7-oxygenated DHEA metabolites after 3 h (*A–C*). Alternatively, cells were incubated with either 7α-hydroxy-, 7β-hydroxy- or 7-keto-DHEA (7-oxo-DHEA) for 24 h, followed by determination of C7-oxygenated DHEA metabolites as described in Materials and Methods (*D*). Data are given as percentage of initially supplied substrate. A representative experiment from three independent transfections is shown. Hatched bars, 7α-hydroxy-DHEA; filled bars, 7β-hydroxy-DHEA, open bars, 7-keto-DHEA.

As shown in Figure 1A, incubation of cells expressing 11β-HSD1 with 1 µM 7α-hydroxy-DHEA led to the formation of both 7β-hydroxy-DHEA and 7-keto-DHEA. Cells co-expressing 11β-HSD1 and H6PDH metabolized less 7α-hydroxy-DHEA, and the accumulation of 7-keto-DHEA was abolished. The fact that 7β-hydroxy-DHEA formation could still be observed suggests that the 7-keto-DHEA formed from 7α-hydroxy-DHEA was rapidly further converted to 7β-hydroxy-DHEA. The involvement of other enzymes in the interconversion of 7-keto- and 7-hydroxy-DHEA in HEK cells is unlikely, since the selective 11β-HSD1 inhibitor T0504 [33], [34] prevented the formation of 7β-hydroxy-DHEA and 7-keto-DHEA, and no conversion was detected in untransfected cells (data not shown). The recovery of the initially added counts was approximately 75%, which was due to a loss of steroids during sampling and during extraction steps. Similarly, incubation of cells expressing 11β-HSD1 with 1 µM 7β-hydroxy-DHEA led to the formation of almost equal amounts of 7-keto-DHEA and 7α-hydroxy-DHEA, while the formation of 7α-hydroxy-DHEA was significantly reduced and that of 7-keto-DHEA almost completely abolished in the presence of H6PDH (Figure 1B). Inhibition of 11β-HSD1 prevented the formation of 7α-hydroxy-DHEA and 7-keto-DHEA.

We then incubated cells expressing 11β-HSD1 with 1 µM 7-keto-DHEA and observed the formation of almost equal amounts of 7α-hydroxy-DHEA and 7β-hydroxy-DHEA (Figure 1C). In the presence of H6PDH, 11β-HSD1 nearly fully metabolized 7-keto-DHEA and preferentially formed 7β-hydroxy-DHEA. A comparison of the products formed after different time intervals indicated that 11β-HSD1 preferentially catalyzes the conversion of 7-keto-DHEA to 7β-hydroxy-DHEA. The formation of 7α-hydroxy-DHEA is less efficient and its accumulation was only observed after prolonged time of incubation. After 24 h of incubation, a steady state was reached and the ratio of 7-keto-DHEA, 7α-hydroxy-DHEA and 7β-hydroxy-DHEA was independent of the substrate chosen initially (Figure 1D). Importantly, the presence of H6PDH strongly shifted the equilibrium from the 7-keto- to the 7β-hydroxy-steroid.

### Analysis of substrate binding by 3D-modelling

To better understand how 7-keto-, 7α-hydroxy- and 7β-hydroxy-DHEA interact with 11β-HSD1, we inserted these steroids into the active site of the recently solved 3D-structure of 11β-HSD1 [35]. In constructing models of DHEA analogs with 11β-HSD1, there are two different orientations in which DHEA analogs can fit in the substrate binding site of 11β-HSD1. In one configuration, the D ring of DHEA is oriented towards the interior of 11β-HSD1. In the other, the A ring is oriented towards the interior.

**Figure 2:**
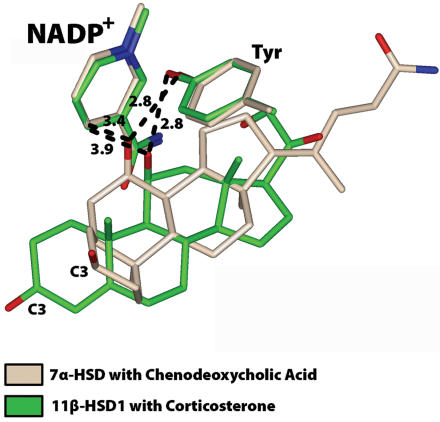
Superposition of *E. coli* 7α-HSD and mouse 11β-HSD1. The 7α-hydroxyl in chenodeoxycholic acid superimposes nicely on the 11β-hydroxyl in corticosterone. The catalytic tyrosine and nicotinamide C4 are favorably positioned to interact with the C7α-hydroxyl and C11β-hydroxyl on chenodeoxycholic acid and corticosterone, respectively. The D rings in chenodeoxycholic acid and corticosterone are oriented towards the interior of 7α-HSD and 11β-HSD1, respectively.

To obtain the first orientation for analysis, we first extracted *E. coli* 7α-HSD from the PDB (file: 1FMC). 7α-HSD contains chenodeoxycholic acid, which has a 7α-hydroxyl group. The D ring of chenodeoxycholic acid is oriented towards the interior of 7α-HSD [36]. We superimposed 1FMC with 1Y5R. As shown in Figure 2, the 7α-hydroxyl in chenodeoxycholic acid and the catalytic tyrosine in 7α-HSD superimpose nicely on the 11β-hydroxyl in corticosterone and the catalytic tyrosine in 11β-HSD1. Initial models of 7-keto-DHEA (with NADPH) and 7β-hydroxy-DHEA (with NADP^+^) with the D ring oriented towards the interior of 11β-HSD1 were constructed by using the Biopolymer option in Insight II for conversion of the 7α-hydroxyl to a ketone and to a 7β-hydroxyl, respectively.

**Figure 3:**
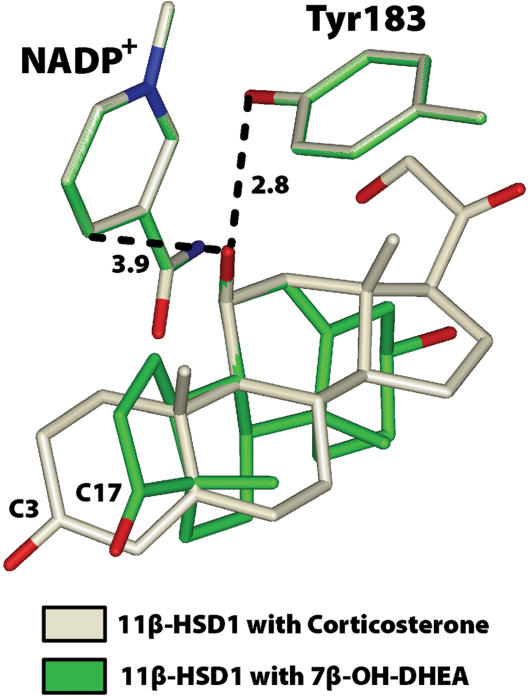
Superposition of 7β-hydroxy-DHEA on corticosterone in 11β-HSD1. In this model, the A ring of 7β-hydroxy-DHEA (7β-OH-DHEA) is oriented towards the interior of 11β-HSD1. The C7β-hydroxyl in 7β-hydroxy-DHEA superimposes nicely on the C11β-hydroxyl in corticosterone with equal predicted distances to C4 of the nicotinamide ring and the hydroxyl of the catalytic tyrosine.

**Figure 4:**
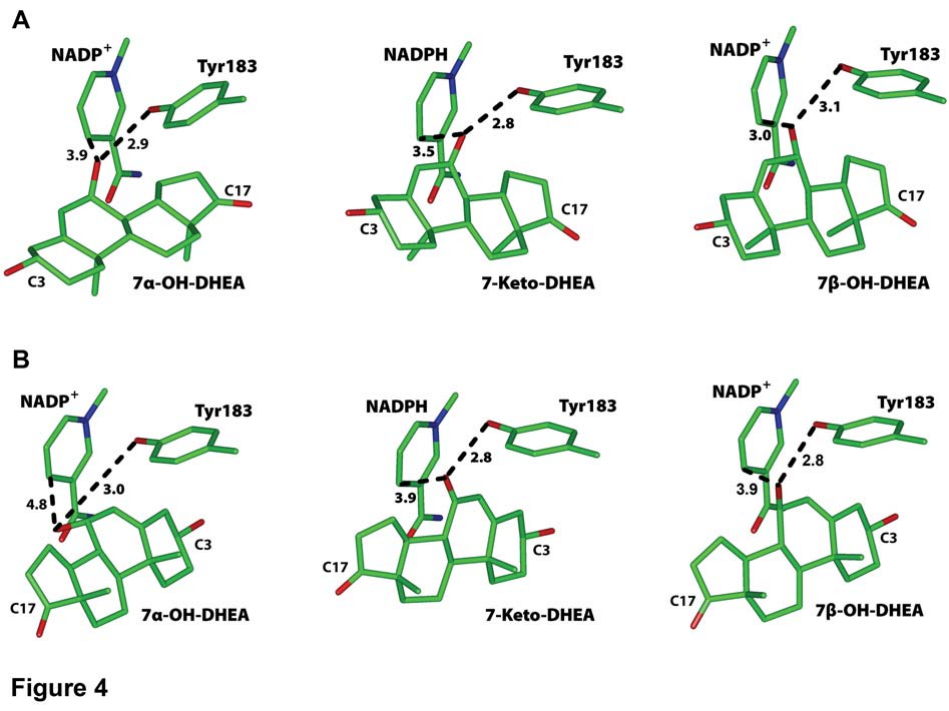
Minimized structures of DHEA analogs in 11β-HSD1. *A*, the D ring is oriented towards the interior of 11β-HSD1. *B*, the A ring is oriented towards the interior of 11β-HSD1.

The second orientation was obtained by overlaying 7β-hydroxy-DHEA with the 11β-hydroxyl on corticosterone in 11β-HSD1 with NADP^+^ (Figure 3). To obtain initial models of 7-keto-DHEA (with NADPH) and 7α-hydroxy-DHEA (with NADP^+^) with the A ring oriented towards the interior of 11β-HSD1, we used the Biopolymer option in Insight II for conversion of the 7β-hydroxyl to a ketone and to a 7α-hydroxyl, respectively. Thus, we had six tertiary complexes of mouse 11β-HSD1 with 7-keto-, 7α-hydroxy- or 7β-hydroxy-DHEA (Figure 4) for refinement by energy minimization using Discover 3.

Figure 4A shows the minimized 3D models of 7-keto-, 7α-hydroxy- or 7β-hydroxy-DHEA with the D ring oriented towards the interior of 11β-HSD1. All of these C7-DHEA analogs have either the hydroxyl or ketone in a favorable position for catalysis by 11β-HSD1. For example, the distance between Tyr-183 and 7α-hydroxyl, 7-keto and 7β-hydroxyl is 2.9 Å, 2.8 Å and 3.1 Å, respectively. The distance between the nicotinamide C4 and 7α-hydroxyl, 7-keto and 7β-hydroxyl is 3.9 Å, 3.5 Å and 3.0 Å, respectively. Figure 4B shows the minimized structures of 7-keto-, 7α-hydroxy- or 7β-hydroxy-DHEA with the A ring oriented towards the interior of 11β-HSD1. 7-keto-DHEA and 7β-hydroxy-DHEA have the ketone and hydroxyl, respectively, in a favorable position for catalysis by 11β-HSD1. In contrast, the 7α-hydroxyl on DHEA is 4.8 Å from the nicotinamide C4.

The models show that 7β-hydroxy-DHEA is favored for reduction when 7-keto-DHEA is in two orientations in 11β-HSD1, while 7α-hydroxy-DHEA is favored in one orientation. Thus, our models can explain the preference for formation of 7β-hydroxy-DHEA by 11β-HSD1.

### Metabolism of other 7-keto steroids by 11β-HSD1

**Figure 5:**
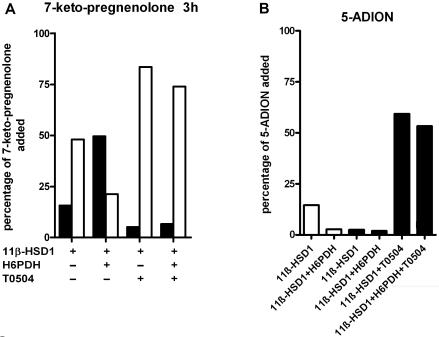
Reduction of 7-ketopregnenolone and 5α-androstane-3β-ol-7,17-dione by 11β-HSD1 in intact cells. HEK-293 cells transfected with a plasmid for 11β-HSD1 and either a control vector or a plasmid for H6PDH were incubated with 1 µM of 7-ketopregnenolone (*A*) or 5α-androstane-3β-ol-7,17-dione (5-ADION) (*B*) in absence or presence of 11β-HSD1 inhibitor T0504, followed by determination of the 7-keto- and 7-hydroxy-metabolites after 3 h (*A,B*) or 6 h (*B*). Data are given as percentage of initially supplied substrate. A representative experiment from three independent transfections is shown. (*A*) 7β-hydroxy-pregnenolone is shown as filled bars and 7-ketopregnenolone as open bars; (*B*) 5α-androstane-3β-ol-7,17-dione (5-ADION) after incubation for 3 h is shown as open bars, and after 6 h as closed bars.

We next investigated whether 11β-HSD1 acts on other 7-keto-steroids and demonstrate for the first time that 11β-HSD1 metabolizes 7-ketopregnenolone and 5α-androstane-3β-ol-7,17-dione (5-ADION)(Figure 5) and that metabolism of both 7-keto-substrates to 7β-hydroxy-steroids was strongly stimulated by H6PDH. For 7-keto-pregnenolone, the equilibrium was shifted from the 7-keto to the 7β-hydroxy form, comparable with the metabolism of 7-keto-DHEA. Since 7α-hydroxypregnenolone and 5α-androstane-3β-ol-7-hydroxy,17-one were not available, the formation of these products could not be analyzed. Peaks in the GC-MS analyses that may correspond to these products were observed and suggest preferential formation of the 7β-hydroxy metabolites (data not shown). The 11β-HSD1-dependent metabolism of 7-ketopregnenolone and 5α-androstane-3β-ol-7,17-dione was almost completely abolished in the presence of inhibitor T0504 (Figure 5), and cells transfected with pcDNA3.1 control plasmid instead of the plasmid for 11β-HSD1 did not show any 7-keto-reductase activity (data not shown).

### Assessment of 7-oxygenated neurosteroids as substrates of 11β-HSD1 by determining competition with glucocorticoids

The modeling analyses indicated that the 7-keto and 7-hydroxy metabolites of DHEA occupy the same binding site in 11β-HSD1 as cortisone and cortisol, respectively. If the 7-keto and 7-hydroxy metabolites of DHEA and pregnenolone are substrates for either the reductase or the dehydrogenase reaction, then they should compete with the reduction of cortisone and the dehydrogenation of cortisol, respectively. Indeed, as shown in Table 1, all three 7-keto-steroids tested preferentially inhibited 11β-HSD1-dependent reduction of cortisone to cortisol with much weaker effects on the reverse reaction. In contrast, 7-hydroxy-steroids preferentially inhibited the dehydrogenase reaction, whereby 7β-hydroxy-DHEA was more potent than 7α-hydroxy-DHEA, in line with the observation that 7β-hydroxy-DHEA is a better substrate for 11β-HSD1 than 7α-hydroxy-DHEA.

**Table 1:** 7-oxygenated neurosteroids compete with 11β-HSD1-dependent cortisone reduction

Compound	11β-HSD1 (oxidation)	11β-HSD1 (reduction)
7-ketodehydroepiandrosterone	29±5	0.82±0.07
7α-hydroxydehydroepiandrosterone	16±1	45±2
7β-hydroxydehydroepiandrosterone	0.54±0.05	7.7±0.8
7-ketopregnenolone	5.4±0.5	0.68±0.11
7β-hydroxypregnenolone	1.34±0.49	2.4±0.5
5α-androstane-3β-ol-7,17dione	18±1	0.50±0.11

11β-HSD activities were determined in lysates of HEK-293 cells expressing recombinant enzyme as described in Materials and Methods. IC_50_ values are in µM. Data represent mean±S.D. from four independent experiments.

### H6PDH stimulates 11β-HSD1 reductase activity and alters the steady state ratio of cortisone to cortisol

Recent studies showed that H6PDH affects 11β-HSD1 function by strongly stimulating the reduction of cortisone and abolishing the oxidation of cortisol [10], [13]. These studies determined only initial rates of conversion. Here, we determined the steady state ratio of cortisone to cortisol in HEK-293 cells transfected with 11β-HSD1 alone or cotransfected with 11β-HSD1 and H6PDH. As shown in Table 2, a steady state ratio of 70–75% cortisol to 25–30% cortisone was reached in cells expressing 11β-HSD1, independent of whether cortisone or cortisol were supplied initially. Co-expression with H6PDH shifted the steady state ratio to 90–95% cortisol and 5–10% cortisone, respectively. Interestingly, the two structurally distinct 11β-HSD1 inhibitors flavanone [34] and BNW7 [37] both had little or no effect on the steady state ratio of glucocorticoids, suggesting that the presence of inhibitors only lowers the time until the equilibrium is reached and that the NADPH availability is the key determinant for the control of the intracellular ratio of cortisone to cortisol. A similar shift from keto- to hydroxy-steroids was observed with 7-substituted steroids (Figure 1 and 5).

**Table 2:** Control of the steady state ratio of cortisone to cortisol by 11β-HSD1 and H6PDH

	*Reduction of cortisone to cortisol*					
	11β-HSD1			11β-HSD1/H6PDH		
	cortisol	cortisone	SD	cortisol	cortisone	SD
Control	71.8%	28.2%	2.6%	94.1%	5.9%	0.3%
Flavanone	74.1%	25.9%	0.7%	91.7%	8.3%	0.3%
BNW7	74.5%	25.5%	1.7%	91.1%	8.9%	0.5%
	*Oxidation of cortisol to cortisone*					
	11β-HSD1			11β-HSD1/H6PDH		
	cortisol	cortisone	SD	cortisol	cortisone	SD
Control	73.1%	26.9%	1.4%	95.4%	4.6%	1.1%
Flavanone	61.2%	39.8%	2.8%	90.9%	9.1%	0.6%
BNW7	73.8%	26.2%	0.7%	90.2%	9.8%	1.3%

The ratio of cortisone to cortisol was measured after incubating HEK-293 cells expressing 11β-HSD1 or coexpressing 11β-HSD1 and H6PDH for 16 h in the presence of 200 nM of radiolabeled substrate. The effect of inhibitors was determined by coincubating cells with 20 µM of the corresponding compound. Data are given as percentage of total glucocorticoid and represent mean±SD, n = 4.

## Discussion

The cloning of 11β-HSD1 has stimulated important advances in understanding its molecular properties and physiological actions. As a result, recombinant 11β-HSD1 in lysates of cells or upon purification became available for determining the kinetic constants for reduction of cortisone and oxidation of cortisol, which revealed that 11β-HSD1 only had a slight preference for reduction of the keto group at C11 on glucocorticoids [2], [3], [38]. In contrast, studies of 11β-HSD1 in whole cells, showed that 11β-HSD1 preferentially was a reductase [4]–[7]. The reaction direction of 11β-HSD1 is crucial with respect to its potential as a target for treatment of metabolic diseases, where a decrease of glucocorticoid reactivation, especially in adipose tissue, is thought to be beneficial [15], [39]. Whether 11β-HSD1 acts as a reductase or dehydrogenase in a given tissue also is important regarding its reported alternative functions. Several studies showed 11β-HSD1-dependent carbonyl reduction of a variety of chemicals, indicating that 11β-HSD1 has a role in detoxification of xenobiotics [16] and oxidized cholesterol [6], [17], [18]. Importantly, it was recently shown that 11β-HSD1 is closely localized to H6PDH in the endoplasmic reticulum [10]. H6PDH provides NADPH for reductases, indicating that the mechanism by which 11β-HSD1 regulates cortisol levels was more complex than previously thought [10], [12], [13].

Here, we provide evidence that in concert with H6PDH, 11β-HSD1 also regulates the levels of 7α-hydroxy-, 7β-hydroxy- and 7keto-neurosteroids derived from DHEA and pregnenolone (Figure 1 and 5). We show that regulation of cosubstrate levels by H6PDH is crucial for determination of the directionality of the synthesis of neurosteroids and glucocorticoids by 11β-HSD1, expanding the physiological functions of 11β-HSD1. We tested various 7-keto- and 7-hydroxy-steroids as substrates of 11β-HSD1 and investigated the influence of H6PDH on their metabolism in intact cells. In line with the results from Muller *et al.* using microsomal fractions from yeast expressing human 11β-HSD1 [22], we detected efficient interconversion of 7-keto- and 7-hydroxy-steroids in an intact human cell system. Importantly, we demonstrate that cells co-expressing 11β-HSD1 and H6PDH predominantly metabolize 7-keto-DHEA, 7-ketopregnenolone and 5α-androstane-3β-ol-7,17-dione into their corresponding 7β-hydroxy metabolites.

The present study provides evidence that 11β-HSD1 plays an important role in regulating the intracellular equilibrium between C7- and C11-keto and their -hydroxy metabolites, respectively. Depending on the level of H6PDH activity, the steady state ratio between keto- and hydroxy-steroids can be controlled. That cosubstrate levels influence how 11β-HSD1 regulates an equilibrium between active versus inactive or less potent steroid hormone metabolites resembles 17β-HSD enzymes. Auchus and coworkers recently showed that at equilibrium 17β-HSD1, 2 and 3 catalyze both reductase and dehydrogenase reactions; thus, these enzymes do not just drive steroid flux in one direction [40], [41].

Our structural modeling provides an explanation for the preferential formation of 7β-hydroxy-DHEA from 7-keto-DHEA. First, the models show that when 7-keto-DHEA, 7α-hydroxy-DHEA and 7β-hydroxy-DHEA have their D ring pointing into the interior of the enzyme, they are in a favorable position to interact with the catalytic tyrosine and the nicotinamide ring (Figure 4A). A similar orientation of the D ring is found for the bile acid chenodeoxycholic acid crystallized in 7α-HSD and corticosterone in 11β-HSD1 (Figure 2). The close superposition of the 7α-hydroxyl on chenodeoxycholic acid and 11β-hydroxyl on corticosterone supports an earlier hypothesis by Lathe, who recognized the rotational symmetry between the 11β- and the 7α-position of the steroid backbone and suggested that some binding sites may recognize both 7α- and 11β-modified steroids [42].

The important discovery from the 3D models is that there are favorable interactions between the C7-hydroxyl and the catalytic site of 11β-HSD1 when the A ring on 7β-hydroxy-DHEA is oriented towards the interior of 11β-HSD1 (Figure 4B). Thus, there are two orientations of 7-keto-DHEA in 11β-HSD1 that can lead to 7β-hydroxy-DHEA in the presence of NADPH, but only one configuration of 7-keto-DHEA is favorable for reduction to 7α-hydroxy-DHEA. This analysis reveals an important role for H6PDH in regulating the specificity for formation of 7β-hydroxy-DHEA. By providing sufficient NADPH, H6PDH can increase cellular levels of 7β-hydroxy-DHEA.

The metabolism of C7-keto and -hydroxy-DHEA and C7-keto and -hydroxy-pregnenolone by 11β-HSD1 and H6PDH can regulate the activities of these neurosteroids in the brain. CYP7B1 is responsible for the production of 7α-hydroxy-DHEA, mainly in the hippocampus [43]. CYP7B1 knock-out mice are unable to produce 7α-hydroxy-DHEA and 7β-hydroxy-DHEA [44], suggesting that 7α-hydroxy-DHEA serves as a precursor for other derivatives including 7β-hydroxy-DHEA and 7-keto-DHEA [28]. The mechanism, however, for the formation of 7β-hydroxy-DHEA and 7-keto-DHEA remained unclear. The present findings indicate that, depending on the activity of H6PDH, 11β-HSD1 can regulate the availability of 7α-hydroxy- 7β-hydroxy- and 7-keto-metabolites of DHEA and pregnenolone in tissues expressing CYP7B1, which can control local concentrations of neurosteroids.

Since cerebrospinal fluid from patients with vascular dementia contained higher levels of 7β-hydroxy-DHEA than that from patients with Alzheimer's disease, Kim *et al.* suggested that 7β-hydroxy-DHEA may be more neuroprotective than 7α-hydroxy-DHEA and that the ratio of 7β-hydroxy-DHEA to 7α-hydroxy-DHEA could be a marker to distinguish between vascular dementia and Alzheimer's disease [45]. Moreover, other 7β-hydroxy-steroids appear to have important protective actions in the brain. For example, Pringle *et al.* reported more efficient reduction of ischemia-induced neuronal damage by 7β-hydroxy-epiandrosterone compared with its 7α-epimer, both *in vivo* and *in vitro* [46].

Interestingly, a single-nucleotide polymorphism associated with a six-fold increased risk for sporadic Alzheimer's disease was recently identified in the promoter region of the *HSD11B1* gene. The polymorphism reduced promoter activity, and the authors suggested that 11β-HSD1 might act as a dehydrogenase in hippocampus and that the polymorphism may result in insufficient inactivation of glucocorticoids with increased neuronal damage [47]. Our data indicate that this polymorphism also will alter levels of 7α-hydroxy- and 7β-hydroxy-DHEA and -pregnenolone, respectively, in the brain, which could contribute to the increased incidence of Alzheimer's disease. A previous study on the distribution of H6PDH activity indicated a ten-fold lower expression in the brain compared with the liver, but the exact localization has not been determined [48]. It will be important to investigate the distribution and activity of H6PDH in specific regions of the brain, with respect to the metabolism of both glucocorticoids and C7-oxygenated steroids by 11β-HSD1.

In conclusion, we provide evidence for a role of 11β-HSD1 in the regulation of the relative ratios of 7α-hydroxy-, 7β-hydroxy- and 7-keto-metabolites of DHEA and pregnenolone, respectively. 11β-HSD1 acts as a reversible enzyme, whereby H6PDH by providing cosubstrate NADPH mediates a shift in the steady state ratio from inactive to active glucocorticoids and from 7α-hydroxy- and 7-keto-steroids to their 7β-hydroxy-forms, respectively. The role of 11β-HSD1 and H6PDH in the metabolism of neurosteroids should be kept in mind when using 11β-HSD1 inhibitors for treatment of patients with the metabolic syndrome.

## Materials and Methods

### Materials

Cell culture media were purchased from Invitrogen (Carlsbad, CA), [1,2,6,7-^3^H]-cortisone from American Radiolabeled Chemicals (St. Louis, MO), [1,2,6,7-^3^H]-cortisol from Amersham Pharmacia (Piscataway, NJ, USA), 5H-1,2,4-triazolo(4,3-a)azepine,6,7,8,9-tetrahydro-3-tricyclo(3·3·1·13·7)dec-1-yl (T0504) from Enamine (Kiev, Ukraine), and reagents for derivatization from Pierce (Rockford, IL). DHEA, 7-keto-DHEA, 7α-hydroxy-DHEA, 7β-hydroxy-DHEA, 5α-androstane-3β-ol-7,17-dione, pregnenolone and 7-ketopregnenolone were obtained from Steraloids (Wilton, NH). BNW7 was kindly provided by Dr. Thomas Wilckens, BioNetWorks GmbH, Munich, Germany. All other chemicals were from Fluka AG (Buchs, Switzerland) of the highest grade available.

### Cell culture and transient transfection

HEK-293 cells were cultured in Dulbecco's Modified Eagle's Medium (DMEM) supplemented with 10% fetal calf serum (FCS), 50 units/ml penicillin, 50 µg/ml streptomycin and 2 mM glutamine. Cells were transfected with expression plasmids for FLAG-tagged 11β-HSD1 and myc-tagged H6PDH or pcDNA3.1 control using the Ca^2+^-phosphate precipitation method. The epitope tags had no effect on enzymatic activities or expression levels of 11β-HSD1 and H6PDH [10], [49]. A total amount of 8 µg DNA was used per 10 cm culture dish or 2 µg DNA per well of a six-well culture plate.

### 11β-HSD activity assays

The enzyme activity in intact cells was measured as described [10]. Briefly, HEK-293 cells, grown in 10 cm culture dishes and transfected with FLAG-tagged 11β-HSD1 alone or co-transfected with myc-tagged H6PDH, were detached 24 h post-transfection and distributed in 96-well plates at a density of 30′000–40′000 cells per well. After 16 h, cells were incubated in serum- and steroid-free medium and the conversion of radiolabeled cortisone to cortisol (or the reverse activity) was determined upon incubation for 1 h at 37°C in a total volume of 50 µl containing 200 nM cortisone (or cortisol). The reaction was stopped by adding methanol containing 2 mM unlabeled cortisone and cortisol, followed by separation of steroids by TLC and scintillation counting. Measurements with freshly prepared cell lysates were carried out in the presence of [1,2,6,7-^3^H]-cortisone and cofactor NADPH at final concentrations of 200 nM and 500 µM for the reductase reaction and with 50 nM [1,2,6,7-^3^H]-cortisol and 500 µM cofactor NAD^+^ for the dehydrogenase reaction, respectively, in the absence or presence of various concentrations of competing 7-oxygenated neurosteroid ligands. The concentration of dimethylsulfoxide or methanol from the solvent was 0.1% in all reactions and had no effect on enzyme activity. Data (mean±SD) were obtained from four independent experiments and were calculated using the Data Analysis Toolbox (Elsevier MDL, Allschwil, Switzerland).

### Measurement of 7-keto-steroid reductase and 7-hydroxy-steroid dehydrogenase activity in intact cells

HEK-293 cells were seeded in six-well plates at a density of 900′000 cells per well. After 24 h, cells were transfected with expression vector for 11β-HSD1 or co-transfected with vectors for 11β-HSD1 and H6PDH. An empty pcDNA3.1 plasmid was used in transfections lacking H6PDH to adjust total DNA amounts in all samples. The medium was replaced 18 h post-transfection by steroid-free medium followed by addition of 1 µM of the steroid substrate to be tested. Compound T0504 at 1 µM was used in control reactions to achieve almost complete inhibition of 11β-HSD1 [33], [34]. After incubation for various time intervals, reactions were terminated by addition of an equal volume of dichloromethane, containing 500 ng medroxyprogesterone as an internal standard. Since radiolabeled C7-oxygenated metabolites were not commercially available, we applied GC-MS for detection. Steroids were extracted and the organic phase separated after centrifugation for 5 min at 2000× g. The organic solvent was evaporated under nitrogen flow, 500 ng of stigmasterol added as an external standard, and the solvent evaporated again. Derivatization was carried out by adding 100 µl of 2% methoxyamine-HCl in pyridine, followed by incubation at 60°C for 1 h. After evaporation of the solvent, 100 µl of trimethylsilylimidazole was added and the samples were incubated for 16 h at 100°C. After cooling to 25°C, the samples were dissolved in 500 µl of cyclohexane∶pyridine∶hexamethyldisilazane (98∶1∶1), and the steroids were purified using Lipidex-500 columns. Finally, steroids were dissolved in 200 µl of cyclohexane, sonicated for 2 min and subjected to GC-MS analysis on a Hewlett Packard gas chromatograph 6890 equipped with a mass-selective detector 5973 by selected ion monitoring.

### 3D-Models of 11β-HSD1

Mouse 11β-HSD1 (PDB ID:1Y5R) was extracted from the Protein Data Bank (PDB) for use as a template to investigate the interactions of 7-keto-, 7α-hydroxy- and 7β-hydroxy-DHEA with 11β-HSD1. We used 1Y5R because it contains both corticosterone and NADP^+^, which allows us to superimpose C7-DHEA analogs on corticosterone. Human 11β-HSD1 (PDB ID: 1XU7) and guinea pig 11β-HSD1 (PDB ID: 1XSE) were co-crystallized with NADP^+^, but not with a glucocorticoid. The close similarity of human and mouse 11β-HSD1, which have 81% sequence identity, allows mouse 11β-HSD1 to be a good model for the interaction of compounds with human 11β-HSD1 [35].

To investigate an alternative, inverted binding mode of 7-keto- and 7-hydroxy-DHEA in 11β-HSD1, we used chenodeoxycholic acid from *E. coli* 7α-hydroxysteroid dehydrogenase (7α-HSD). Chenodeoxycholic acid is an excellent template because it has a 7α-hydroxyl. We superimposed the PDB file 1FMC (*E. coli* 7α-HSD with chenodeoxycholic acid) with 1Y5R and extracted chenodeoxycholic acid, which was merged into 11β-HSD1. For conversion of the 7α-hydroxyl to a ketone or a 7β-hydroxyl we used the Biopolymer option in Insight II. The energy of each model of 11β-HSD1 with C7-DHEA analogs was minimized using Discover 3 (Accelrys Inc., San Diego, CA, USA), which was run for 10,000 iterations, using a distant dependent dielectric constant of 2.

## References

[1] Agarwal AK, Monder C, Eckstein B, White PC (1989). Cloning and expression of rat cDNA encoding corticosteroid 11 beta-dehydrogenase.. J Biol Chem.

[2] Agarwal AK, Tusie-Luna MT, Monder C, White PC (1990). Expression of 11 beta-hydroxysteroid dehydrogenase using recombinant vaccinia virus.. Mol Endocrinol.

[3] Nobel CS, Dunas F, Abrahmsen LB (2002). Purification of full-length recombinant human and rat type 1 11beta-hydroxysteroid dehydrogenases with retained oxidoreductase activities.. Protein Expr Purif.

[4] Jamieson PM, Walker BR, Chapman KE, Andrew R, Rossiter S (2000). 11 beta-hydroxysteroid dehydrogenase type 1 is a predominant 11 beta-reductase in the intact perfused rat liver.. J Endocrinol.

[5] Draper N, Walker EA, Bujalska IJ, Tomlinson JW, Chalder SM (2003). Mutations in the genes encoding 11beta-hydroxysteroid dehydrogenase type 1 and hexose-6-phosphate dehydrogenase interact to cause cortisone reductase deficiency.. Nat Genet.

[6] Schweizer RA, Zurcher M, Balazs Z, Dick B, Odermatt A (2004). Rapid hepatic metabolism of 7-ketocholesterol by 11beta-hydroxysteroid dehydrogenase type 1: species-specific differences between the rat, human, and hamster enzyme.. J Biol Chem.

[7] Apostolova G, Schweizer RA, Balazs Z, Kostadinova RM, Odermatt A (2005). Dehydroepiandrosterone inhibits the amplification of glucocorticoid action in adipose tissue.. Am J Physiol Endocrinol Metab.

[8] Bujalska IJ, Walker EA, Hewison M, Stewart PM (2002). A switch in dehydrogenase to reductase activity of 11 beta-hydroxysteroid dehydrogenase type 1 upon differentiation of human omental adipose stromal cells.. J Clin Endocrinol Metab.

[9] Lavery GG, Walker EA, Draper N, Jeyasuria P, Marcos J (2006). Hexose-6-phosphate dehydrogenase knock-out mice lack 11 beta-hydroxysteroid dehydrogenase type 1-mediated glucocorticoid generation.. J Biol Chem.

[10] Atanasov AG, Nashev LG, Schweizer RA, Frick C, Odermatt A (2004). Hexose-6-phosphate dehydrogenase determines the reaction direction of 11beta-hydroxysteroid dehydrogenase type 1 as an oxoreductase.. FEBS Lett.

[11] Frick C, Atanasov AG, Arnold P, Ozols J, Odermatt A (2004). Appropriate function of 11beta-hydroxysteroid dehydrogenase type 1 in the endoplasmic reticulum lumen is dependent on its N-terminal region sharing similar topological determinants with 50-kDa esterase.. J Biol Chem.

[12] Banhegyi G, Benedetti A, Fulceri R, Senesi S (2004). Cooperativity between 11beta-hydroxysteroid dehydrogenase type 1 and hexose-6-phosphate dehydrogenase in the lumen of the endoplasmic reticulum.. J Biol Chem.

[13] Bujalska IJ, Draper N, Michailidou Z, Tomlinson JW, White PC (2005). Hexose-6-phosphate dehydrogenase confers oxo-reductase activity upon 11 beta-hydroxysteroid dehydrogenase type 1.. J Mol Endocrinol.

[14] Hewitt KN, Walker EA, Stewart PM (2005). Minireview: hexose-6-phosphate dehydrogenase and redox control of 11{beta}-hydroxysteroid dehydrogenase type 1 activity.. Endocrinology.

[15] Atanasov AG, Odermatt A (2007). Readjusting the glucocorticoid balance: An opportunity for modulators of 11b-hydroxysteroid dehydrogenase type 1 activity?. Endocr Metab Immune Disord Drug Targets.

[16] Maser E, Wsol V, Martin HJ (2006). 11Beta-hydroxysteroid dehydrogenase type 1: purification from human liver and characterization as carbonyl reductase of xenobiotics.. Mol Cell Endocrinol.

[17] Song W, Chen J, Dean WL, Redinger RN, Prough RA (1998). Purification and characterization of hamster liver microsomal 7alpha-hydroxycholesterol dehydrogenase. Similarity to type I 11beta-hydroxysteroid dehydrogenase.. J Biol Chem.

[18] Hult M, Elleby B, Shafqat N, Svensson S, Rane A (2004). Human and rodent type 1 11beta-hydroxysteroid dehydrogenases are 7beta-hydroxycholesterol dehydrogenases involved in oxysterol metabolism.. Cell Mol Life Sci.

[19] Muller C, Hennebert O, Morfin R (2006). The native anti-glucocorticoid paradigm.. J Steroid Biochem Mol Biol.

[20] Robinzon B, Michael KK, Ripp SL, Winters SJ, Prough RA (2003). Glucocorticoids inhibit interconversion of 7-hydroxy and 7-oxo metabolites of dehydroepiandrosterone: a role for 11beta-hydroxysteroid dehydrogenases?. Arch Biochem Biophys.

[21] Robinzon B, Prough RA (2005). Interactions between dehydroepiandrosterone and glucocorticoid metabolism in pig kidney: Nuclear and microsomal 11beta-hydroxysteroid dehydrogenases.. Arch Biochem Biophys.

[22] Muller C, Pompon D, Urban P, Morfin R (2006). Inter-conversion of 7alpha- and 7beta-hydroxy-dehydroepiandrosterone by the human 11beta-hydroxysteroid dehydrogenase type 1.. J Steroid Biochem Mol Biol.

[23] Miller WL (2002). Androgen biosynthesis from cholesterol to DHEA.. Mol Cell Endocrinol.

[24] Baulieu EE, Robel P (1998). Dehydroepiandrosterone (DHEA) and dehydroepiandrosterone sulfate (DHEAS) as neuroactive neurosteroids.. Proc Natl Acad Sci U S A.

[25] Compagnone NA, Mellon SH (1998). Dehydroepiandrosterone: a potential signalling molecule for neocortical organization during development.. Proc Natl Acad Sci U S A.

[26] Wolf OT, Kirschbaum C (1999). Actions of dehydroepiandrosterone and its sulfate in the central nervous system: effects on cognition and emotion in animals and humans.. Brain Res Brain Res Rev.

[27] Matsunaga M, Ukena K, Baulieu EE, Tsutsui K (2004). 7alpha-Hydroxypregnenolone acts as a neuronal activator to stimulate locomotor activity of breeding newts by means of the dopaminergic system.. Proc Natl Acad Sci U S A.

[28] Lardy H, Partridge B, Kneer N, Wei Y (1995). Ergosteroids: induction of thermogenic enzymes in liver of rats treated with steroids derived from dehydroepiandrosterone.. Proc Natl Acad Sci U S A.

[29] Yau JL, Rasmuson S, Andrew R, Graham M, Noble J (2003). Dehydroepiandrosterone 7-hydroxylase CYP7B: predominant expression in primate hippocampus and reduced expression in Alzheimer's disease.. Neuroscience.

[30] Weill-Engerer S, David JP, Sazdovitch V, Liere P, Schumacher M (2003). In vitro metabolism of dehydroepiandrosterone (DHEA) to 7alpha-hydroxy-DHEA and Delta5-androstene-3beta,17beta-diol in specific regions of the aging brain from Alzheimer's and non-demented patients.. Brain Res.

[31] Hampl R, Morfin R, Starka L (1997). Minireview 7-Hydroxylated Derivatives Of Dehydroepiandrosterone: What Are They Good For?. Endocr Regul.

[32] Shi J, Schulze S, Lardy HA (2000). The effect of 7-oxo-DHEA acetate on memory in young and old C57BL/6 mice.. Steroids.

[33] Hermanowski-Vosatka A, Balkovec JM, Cheng K, Chen HY, Hernandez M (2005). 11beta-HSD1 inhibition ameliorates metabolic syndrome and prevents progression of atherosclerosis in mice.. J Exp Med.

[34] Arampatzis S, Kadereit B, Schuster D, Balazs Z, Schweizer RA (2005). Comparative enzymology of 11beta-hydroxysteroid dehydrogenase type 1 from six species.. J Mol Endocrinol.

[35] Zhang J, Osslund TD, Plant MH, Clogston CL, Nybo RE (2005). Crystal Structure of Murine 11beta-Hydroxysteroid Dehydrogenase 1: An Important Therapeutic Target for Diabetes.. Biochemistry.

[36] Tanaka N, Nonaka T, Tanabe T, Yoshimoto T, Tsuru D (1996). Crystal structures of the binary and ternary complexes of 7 alpha-hydroxysteroid dehydrogenase from Escherichia coli.. Biochemistry.

[37] Schuster D, Maurer EM, Laggner C, Nashev LG, Wilckens T (2006). The discovery of new 11beta-hydroxysteroid dehydrogenase type 1 inhibitors by common feature pharmacophore modeling and virtual screening.. J Med Chem.

[38] Arnold P, Tam S, Yan L, Baker ME, Frey FJ (2003). Glutamate-115 renders specificity of human 11beta-hydroxysteroid dehydrogenase type 2 for the cofactor NAD(+).. Mol Cell Endocrinol.

[39] Wang M (2006). Inhibitors of 11beta-hydroxysteroid dehydrogenase type 1 for the treatment of metabolic syndrome.. Curr Opin Investig Drugs.

[40] Khan N, Sharma KK, Andersson S, Auchus RJ (2004). Human 17beta-hydroxysteroid dehydrogenases types 1, 2, and 3 catalyze bi-directional equilibrium reactions, rather than unidirectional metabolism, in HEK-293 cells.. Arch Biochem Biophys.

[41] Agarwal AK, Auchus RJ (2005). Minireview: cellular redox state regulates hydroxysteroid dehydrogenase activity and intracellular hormone potency.. Endocrinology.

[42] Lathe R (2002). Steroid and sterol 7-hydroxylation: ancient pathways.. Steroids.

[43] Chalbot S, Trap C, Monin JP, Morfin R (2002). Use of bioconversion for the preparation of [4-14C]-labeled 7 alpha- and 7 beta-hydroxylated derivatives of dehydroepiandrosterone and epiandrosterone.. Steroids.

[44] Rose K, Allan A, Gauldie S, Stapleton G, Dobbie L (2001). Neurosteroid hydroxylase CYP7B: vivid reporter activity in dentate gyrus of gene-targeted mice and abolition of a widespread pathway of steroid and oxysterol hydroxylation.. J Biol Chem.

[45] Kim SB, Hill M, Kwak YT, Hampl R, Jo DH (2003). Neurosteroids: Cerebrospinal fluid levels for Alzheimer's disease and vascular dementia diagnostics.. J Clin Endocrinol Metab.

[46] Pringle AK, Schmidt W, Deans JK, Wulfert E, Reymann KG (2003). 7-Hydroxylated epiandrosterone (7-OH-EPIA) reduces ischaemia-induced neuronal damage both in vivo and in vitro.. Eur J Neurosci.

[47] de Quervain DJ, Poirier R, Wollmer MA, Grimaldi LM, Tsolaki M (2004). Glucocorticoid-related genetic susceptibility for Alzheimer's disease.. Hum Mol Genet.

[48] Tanahashi K, Hori SH (1980). Immunohistochemical localization of hexose 6-phosphate dehydrogenase in various organs of the rat.. J Histochem Cytochem.

[49] Odermatt A, Arnold P, Stauffer A, Frey BM, Frey FJ (1999). The N-terminal anchor sequences of 11beta-hydroxysteroid dehydrogenases determine their orientation in the endoplasmic reticulum membrane.. J Biol Chem.

